# Refugee blues: a UK and European perspective

**DOI:** 10.3402/ejpt.v6.29328

**Published:** 2015-10-28

**Authors:** Stuart Turner

**Affiliations:** Trauma Clinic, London, United Kingdom

**Keywords:** Asylum, refugees, deterrence, decision making

## Abstract

In this paper, the numbers of refugees travelling to the European Union are set in a global context. It is argued that the increasing restrictions placed on asylum seekers from the 1980s onwards in the UK and the associated culture of deterrence and prohibition have had the perverse effect of supporting the economic market for people smuggling. It appears that these restrictions were initially designed to deter people, most of whom would have been granted humanitarian assistance had they managed to arrive in the UK, so as to prevent them from accessing the decision-making process on asylum. Policy changes concerning travel, benefits, and other pressures on asylum seekers are also considered in the context of deterrence. The problems facing asylum seekers do not end with their arrival in a safe country. The current methods of determining refugee status are alarmingly weak. Indeed there is evidence suggesting that those who are most traumatised before arrival face systematic disadvantage. The focus of this paper is on the United Kingdom but its conclusions apply to most Western European countries. The paper concludes with some tentative suggestions for change.

Recent news stories have highlighted the plight of refugees travelling into Western Europe across the Central Mediterranean and, more recently, across the Eastern Mediterranean and through Hungary. Frontex (the European Agency for the Management of Operational Cooperation at the External Borders of the Member States of the European Union) estimates that in 2012, 15,151 migrants travelled across the Central Mediterranean; these numbers grew to 45,298 in 2013 and 170,664 in 2014 (Frontex, 2015). The United Nations High Commission for Refugees (UNHCR) has reported that Europe is living through a “maritime refugee crisis” of historic proportions (UNHCR, 2015a). It asserts that the majority of migrants taking the sea route are refugees, eligible for protection. For example, in the first half of 2015, 43,900 Syrians arrived in Western Europe by sea (and UNHCR observes that in 2014, 95% of Syrians after arrival were acknowledged in Europe as refugees).

They are often transported across the Mediterranean by people smugglers—in boats that are not fit for the journey, overcrowded, and frankly dangerous. Following the death of 368 refugees off Lampedusa in October 2013, a search and rescue operation (Mare Nostrum) was launched. This was scaled back in December 2014. UNHCR reports that in the first 3 months of 2015, 479 refugees and migrants drowned or went missing, as opposed to 15 in the first 3 months of 2014. In April 2015, 1,308 refugees and migrants drowned or went missing in a single month (UNHCR, 2015a).

People smuggling is simply defined as the procurement for financial or other material benefit of the illegal entry of a person into a state of which that person is not a national or resident (United Nations, 2004), an activity carried out for personal gain. It is to be distinguished from the many examples of altruistic behaviour, aimed at helping people cross frontiers. The recent death of Sir Nicholas Winton, for example, reminds us of those humanitarians involved in the “kindertransport” programme in the period leading up to WWII (Guardian, 2015). People smuggling is a commercial activity subject to economic forces of supply and demand.

At first sight, the problem appears straightforward, and this is how it is often portrayed in the media. Those who are “bogus,” simply seeking financial betterment in Europe should be discouraged or promptly returned, whereas “genuine refugees” should be provided with humanitarian assistance in what has been called the “proud tradition” of European generosity. If only it were so simple.

Following WWII, there was a recognition that, within the framework of the Universal Declaration of Human Rights (United Nations, 1948), it was important to protect the rights of refugees. The 1951 Convention Relating to the Status of Refugees (United Nations, 2010) defines a refugee as someone who, owing to a well-founded fear of being persecuted for reasons of race, religion, nationality, membership of a particular social group or political opinion, is outside the country of his nationality and is unable to or, owing to such fear, is unwilling to avail himself of the protection of that country. The convention was initially designed to respond to the needs of Europeans displaced in the war, but the 1967 Protocol removed these temporal and geographical restrictions (United Nations, 2010).

The United Nations High Commissioner for Refugees, in an introductory note to the Refugee Convention at the time of its 60th anniversary (United Nations, 2010) emphasises that it is “under-pinned by a number of fundamental principles.” For example, “non-refoulement” is considered to be so fundamental that no reservations or derogations may be made to it. It provides that, “no one shall expel or return (‘refouler’) a refugee against his or her will, in any manner whatsoever, to a territory where he or she fears threats to life or freedom.” The convention also stipulates that, subject to specific exceptions, refugees “should not be penalised for their illegal entry or stay.” It also recognises that the process of seeking asylum “can require refugees to breach immigration rules.”

There has been increasing concern about the apparently chaotic movement of refugees from one country to another within the European Union, partly procedural and partly political. This probably came to a head on 2 September 2015, when photographs of a Syrian toddler, drowned and lying on a tourist beach triggered public outrage and a further review of the European Union's internal policies on migration. Although some European countries have agreed a plan for allocation by quota, this has yet to be fully implemented. The Dublin Regulation has generally provided a framework designed to ensure that only one European country is responsible for examining an asylum application. The criteria for establishing responsibility run, in hierarchical order, from family considerations, to recent possession of visa or residence permit in a European country, to whether the applicant has entered EU irregularly, or regularly (European Commission, 2015). A factsheet from the European Court of Human Rights (ECHR), however, reveals some of the difficulties in its implementation (ECHR, 2015); this regulation was last updated on 1 January 2014.

This all sounds very good, but in practice, implementing the Refugee Convention, at least in Europe, has proved to be increasingly difficult—largely to do with the numbers potentially involved and a failure of political leadership in the face of media hostility to migrants in general.

## How many of the world's refugees travel to Western Europe?

UNHCR publishes regular reports concerning the numbers of people worldwide seen as being of concern to the Agency. At the end of 2014, it estimated that there were 59.5 million forcibly displaced people worldwide (UNHCR, 2015b). This included 19.5 million refugees, around 2.9 million more than in 2013. There were also about 38.2 million internally displaced people (IDPs).

**Fig. 1:**
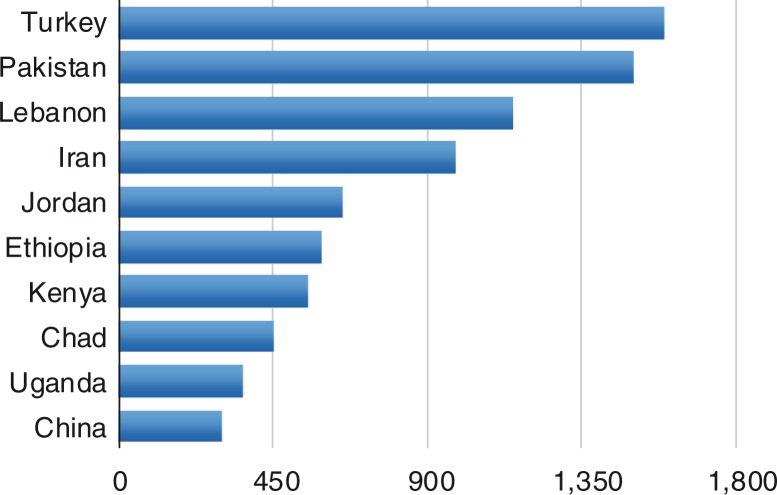
Top 10 host countries, end 2014 (in thousands). Source: UNHCR, 2015b.

UNHCR estimates that 86% of the world's refugees live in developing countries (compared with 70%, 10 years ago). By the end of 2014, Syria had become the world's top source country for refugees, overtaking Afghanistan, which had held this position for over three decades. Despite the stereotypes so common in the media, relatively few refugees find their way to Western Europe. There are different ways of looking at the data (UNHCR, 2015b). Simply looking at raw numbers (Fig. 1), the main receiving country in 2014 was Turkey, overtaking Pakistan. Of the world's refugees, UNHCR estimates that 51% are under the age of 18, the highest figure in more than a decade.

**Fig. 2:**
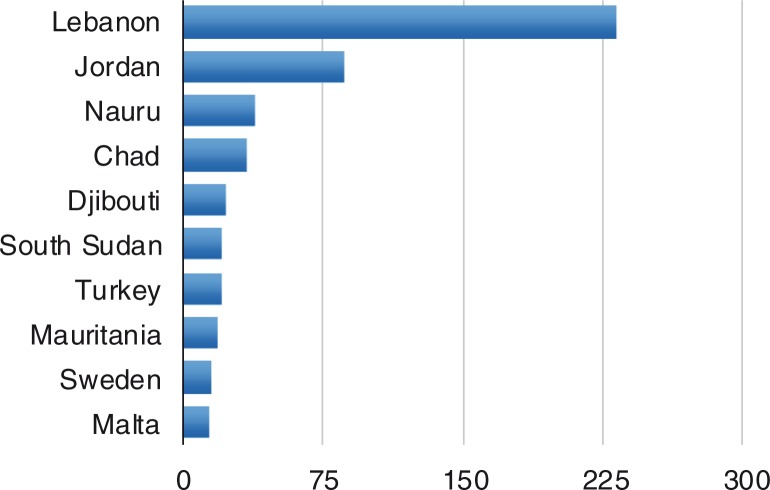
Refugees per 1,000 inhabitants. Source: UNHCR, 2015b.

Displaying the data with reference to the size of the host population (Fig. 2) demonstrates the extraordinary impact of the Syrian conflict; Turkey registered over 1 million Syrian refugees in 2014 alone and Lebanon registered over 400,000 Syrian refugees. Turkey, Pakistan, Lebanon, and Iran hosted 5.2 million of all refugees between them, amounting to 36% of all refugees worldwide.

**Fig. 3:**
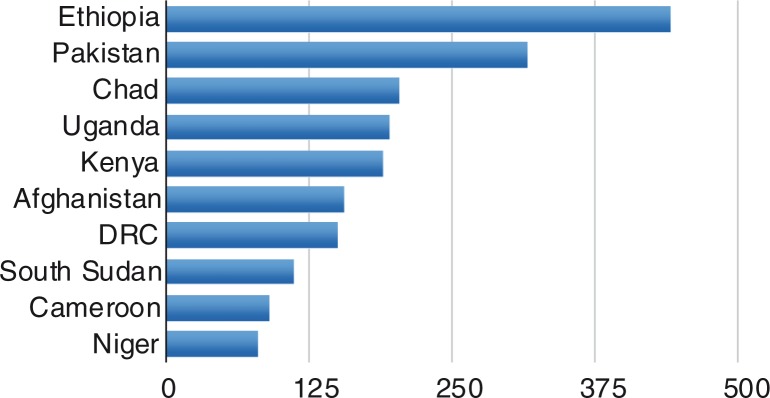
Refugees per 1 US$ GDP (PPP) per capita. Source: UNHCR, 2015b.

UNHCR also provides a measure of impact taking into account per capita income of the country (Fig. 3). This illustrates the financial impact on countries such as Ethiopia, Afghanistan, DRC, and South Sudan.

So the data clearly indicate that most refugees find their way to neighbouring, often relatively poor countries, rather than to Western Europe.

## United Kingdom: a case example

**Fig. 4:**
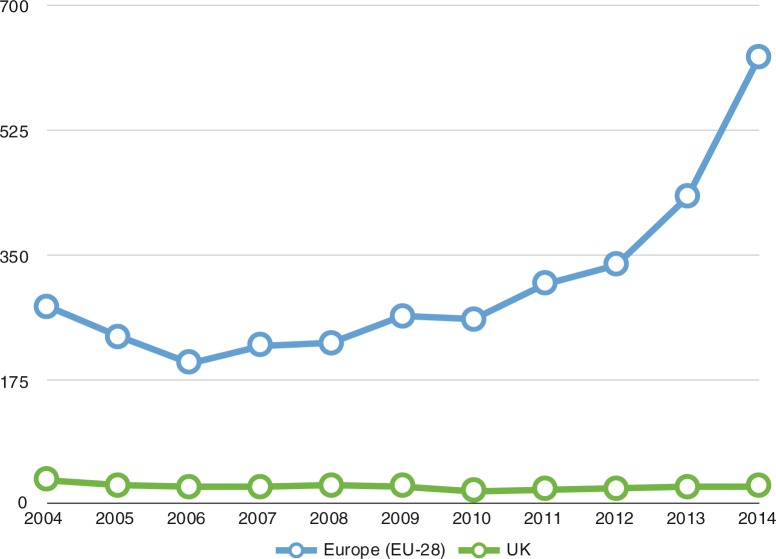
Asylum claims in the UK and Europe (in thousands). Sources: UK Office for National Statistics (www.ons.gov.uk/ons/rel/migration1/migration-statistics-quarterly-report/february-2015/stb-msqr-feb-2015.html and www.ons.gov.uk/ons/dcp171778_335330.pdf) and Eurostat (www.ec.europa.eu/eurostat/statistics-explained/index.php/Asylum_statistics).

In this paper, the focus will be on the UK. This is not because it is necessarily better or worse than other European countries but simply because the situation in the UK is better known to the author. To put the UK data in context, in 2014 Germany received 202,645 asylum seekers, Sweden received 81,180, Italy received 64,625, France received 64,310, Hungary received 42,775, whereas the UK received only 31,745 (Eurostat, 2015). Indeed, by comparison with other European countries, the UK has received remarkably few refugees (Fig. 4).

**Fig. 5:**
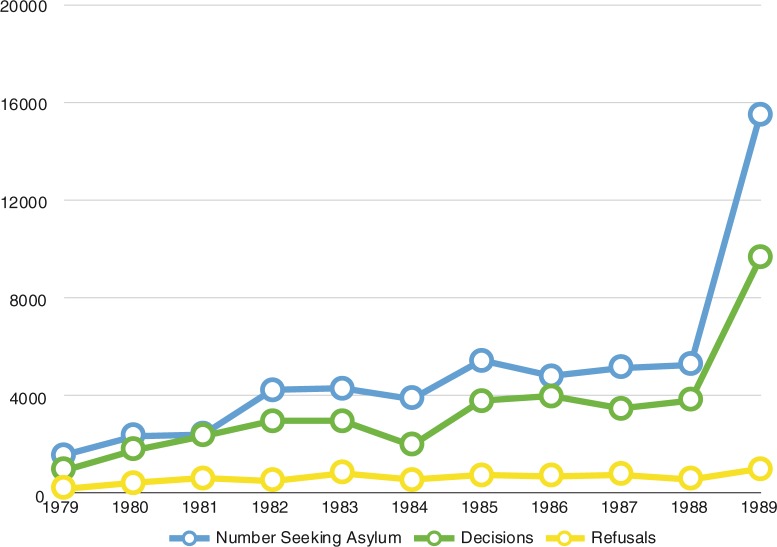
Applications for refugee status and asylum in the UK (1979–1989). Source: Home office, 1990.

Thirty years ago, in the 1980s, the UK was receiving around 4,000–5,000 asylum seekers a year (Fig. 5). It was at the end of that decade that the numbers began to increase significantly. For this reason alone, the period is worth examining carefully.

Over these 10 years, there was a change in the profile of asylum seekers arriving in the UK. Whereas in 1979, the largest group of asylum seekers came from Iran (following its revolution that year), by 1989, the largest number of asylum seekers were from Turkey (Home Office, 1990). There were also important changes in UK policy. The British Nationality Act (1981) set out to tighten the criteria for UK citizenship. In 1985, new visa restrictions were introduced for Sri Lanka (following the increase in violence there in July 1983). The Carriers Liability Act of 1987 penalised carriers, especially international airlines, if they transported people to the UK without valid documentation, thus ensuring that carriers were careful to check documents before embarkation. In 1989, new visa restrictions were introduced for Turkey. In other words, policy was directed at deterring or preventing potential asylum seekers from arriving in the UK. This would be understandable if it was clear that the system was being widely abused. Yet the data did not support this contention (Fig. 5). The percentage of people refused humanitarian support fell in this decade from 22% in 1979 to 10% in 1989 (Home Office, 1990).

It is true that fewer and fewer were granted full refugee status and correspondingly more were given exceptional leave to remain (ELR) on humanitarian grounds (Home Office, 1990). Although in practice, many of those given ELR were able to remain permanently, their official status was temporary and subject to review. It was an uncertain time for those people who were genuinely afraid of being returned to their own countries. Although a reasonable response where circumstances in the country of origin were seen as temporary, in general this was probably also part of a deterrent policy rather than due to a real change in the circumstance of applicants.

**Fig. 6:**
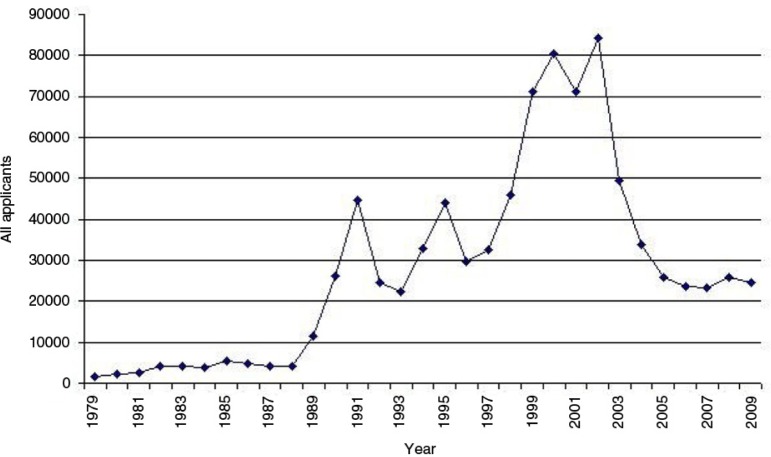
UK Asylum applications 1979–2009. Source: www.commons.wikimedia.org/wiki/File:UK_asylum_applications_1979–2009.jpg

So during this decade, although it became harder for refugees to travel to the UK and seek asylum there, the numbers arriving increased. This was almost certainly because of a growth in the use of “agents” to assist asylum seekers in their journeys. Many did this from compassion, from an altruistic, humanitarian, impulse. However, others began to realise that this was a potentially lucrative business with a ready market. So just as with any other policy of prohibition, these deterrent policies probably had the unintended effect of facilitating an underground market in people smuggling. From 1989 to 2002 (Fig. 6), there were further large increases in numbers of asylum seekers (Blinder, 2014).

Although perhaps this just reflected the European position as a whole (Blinder, 2014), in response, UK policy and legislative changes were directed to try and find tougher means to deter entry. In January 2002, a UK government minister was asked in parliament if there were any legal means by which an individual could enter the UK and claim asylum; the short answer he gave was “no” (House of Lords Hansard, 2002).

There were also policies to make the process of seeking asylum less attractive. The 1999 Immigration and Asylum Act introduced a dispersal policy. Ostensibly this was to relieve pressure on the southeast of England and to spread the burden of asylum seekers more widely across the UK. However, this has also been seen as a mechanism of exclusion, part of a policy of deterrence (Stewart, 2012). Since 2000, asylum seekers have been housed on a no-choice basis at locations around the country. The selection of dispersal locations rather than being guided by the presence of existing ethnic communities has been largely driven by available housing. So they are generally concentrated in socially deprived areas.

Changes to the benefits regime probably also had the intention of making the UK appear less attractive to asylum seekers. In a report for Oxfam and the Refugee Council, Penrose (2002) found that 85% of those organisations surveyed reported that their clients experienced hunger and 95% that their clients were unable to afford clothes or shoes. The support received by asylum seekers was set at less than that available as “the minimum level of income required to maintain an acceptable standard of living” to UK residents. Penrose goes on to suggest that the “Government justifies its current asylum policy and the establishment of a separate system of support on the grounds that many ‘bogus’ asylum-seekers come to this country to live off the state and that providing a lower level of support will help to deter individual claims for asylum. In fact there is little evidence to support such an argument.” Under the 2002 Nationality, Immigration and Asylum Act, only people making applications at ports as soon as reasonably practicable were to be eligible for benefits—basic accommodation and financial support. In March 2003, the appeal court agreed that this would be in breach of the European Human Rights Convention, leaving asylum seekers potentially destitute and at risk of inhumane and degrading treatment (Guardian, 2003).

The Asylum and Immigration Act 1996 set in place a new criminal sanction against employers taking on people without permission to work in the UK, including many asylum seekers. This probably provided a further deterrent pressure, adding to the effects of changes in the benefit system and perhaps pushing some people into illegal working—without pay or other protections available to other employees.

Between 2001 and 2004, it is said that Prime Minister Blair probably held more meetings to discuss the issue of asylum than any other issue except Iraq. Spencer (2009) suggests that, “Blair's overriding objective was to convince the public that migration was under control and to neutralise immigration as a political issue. In that, he undoubtedly failed, polls showing public concern rising throughout his period in office.”

Numbers of asylum seekers did fall after 2002, but the policy of deterrence continued—for example, with a growth in numbers detained for immigration purposes over 30 years from 1,304 in 1980 to 28,005 in 2010 (Institute of Race Relations, 2015). Detention is not without risk. In 2012, for example, it was reported that there were 208 incidents of self-harm requiring medical attention in immigration detention and 1,804 incidences where detainees were put on formal self-harm risk monitoring in this period (No-deportations, 2013). There is no good estimate of the full emotional impact of this form of detention in the UK, although Silove, Steel, and Watters (2000) appropriately highlight the potential harm from post-migration stress in traumatised refugees and the need to educate governments and the public about the potential risks of harsh policies of deterrence.

An analysis of the justifications and counterarguments used in parliament led Fletcher (2008) to conclude that, “The paucity of references to statistics and evidence in the political discourse reveals how ideas gain ground not through reasoned discussions informed by empirical research, but through the expounding of ideologies, rhetoric and persuasion.” It is suggested that those (here described as “partialists”), who emphasise the importance of the links between the state and its citizens, seeing states as under no obligation to anyone outside their citizenship and morally justified in prioritising and privileging their own citizens over outsiders, have been increasingly dominant in Europe.

## Why do people travel to Europe?

In the face of a policy of deterrence, it is reasonable to ask why people do travel to Europe (and, in the context of this paper, specifically to Britain) to seek asylum. Neumayer (2005a) has undertaken extensive research on European data, looking at the factors that drive migration from other countries. He concludes that economic conditions in countries of origin are statistically significant and substantively important determinants of aggregate asylum migration to Western Europe. This implies that policies aimed at improving economic conditions in these countries, such as development assistance and the opening of protected European markets to imports from developing countries, can lower the migration pressure from these countries. According to Neumayer, a one percent increase in per capita income reduces numbers of asylum seekers to Europe by one percent. At the same time, his results “clearly demonstrate the importance of the political regime in origin countries and of threats to the personal integrity of individuals from human rights abuse, dissident political violence, civil/ethnic warfare and state failure as well as possibly external conflict.” Autocracy, in particular, appeared to be an important cause of migration. Neumayer argues that, “If Western European countries want to tackle the root causes of asylum migration, then they need to undertake policy measures that promote economic development, democracy, respect for human rights and peaceful conflict resolution in countries of origin.” This is a position likely to be accepted by those who emphasise the importance of human rights, but even those most closely embracing the “partialist” position with regard to accepting asylum seekers into the UK should also consider the political advantages of endorsing this approach to foreign policy.

Within Europe, some countries appear more popular than others (at different times). Research studies undertaken separately on behalf of the UK government (Robinson & Segrott, 2002) and the Refugee Council (Crawley, 2010), both suggest that the decision to choose the UK over other countries, for example, is often not made by the asylum seeker but by others involved in their transportation. In the latter study, the single most important reason for arrival in the UK was that a decision had been made by others. Both studies emphasise the increasing importance of agents. This suggests that the market in people smuggling—whether across the Mediterranean or into the UK is a major factor determining country of destination. This is important evidence to take into account in policy determination.

In the Mediterranean, Mare Nostrum (Ministero della Difesa, 2015), the search and rescue operation carried out by the Italian Navy since the deaths of hundreds of migrants off Lampedusa in October 2013, was formally closed on 1 November 2014 (having rescued over 150,000 migrants at high sea) and was replaced by operation Triton, a border control operation (European Council on Refugees and Exiles, 2014). One of the reasons for the decision was said to be that rescuing people in the Mediterranean would encourage more to attempt the journey. In a public opinion survey in April 2015, in Great Britain, 55% believed that search and rescue would encourage immigration, compared with 26% who did not. Even in Germany, the most tolerant of the nations surveyed, 44% believed it would favour immigration as opposed to 37% who did not (YouGov, 2015).

However, since the trade in moving people across the Mediterranean was probably driven by people smugglers (paid in advance for their services and usually not facing risk of death in transit themselves) rather than refugees themselves, it was probably naive to expect that the ending of Mare Nostrum would achieve a reduction in migration in the short term. Indeed, as UNHCR (2015a) has reported, what was achieved was a dramatic increase in deaths, 1,308 drowned or missing in April 2015 alone. Desperate people travelling from countries like Syria will be prepared to take risks; those doing the smuggling will be better placed to assess the degree of risk but the profit motive will stand to continue to drive this activity regardless of the likelihood of naval rescue at sea.

In summary, it is likely that increasing use of prohibition tactics, closing down legal routes of entry, has had the unintended perverse result of favouring those running clandestine routes. People smuggling has become a business and those profiting from it will have their own reasons for increasing demand. Western policies of deterrence lead to more and more draconian steps being enacted to prevent access (of people, many of whom would receive humanitarian protection if they were able to travel) and therefore the growth in potential profits for people smugglers.

## For those who do arrive …

Simply arriving in a Western country, such as the UK, is not the end to the difficulties that many face. The process of determination of refugee status is also fraught with problems. This is partly because it is such a tough decision to make. The decision relies on two main factors. The first concerns the nature of the claim being made and a view as to whether it satisfies one of the legal tests; this is relatively straightforward. However, the second is to do with the credibility of the claim; this is an area where decision making becomes especially subjective and therefore amenable to influence by external factors (or even by the emotions of the decision-maker at the time). Thus in Europe, Neumayer (2005b) found that recognition rates for full refugee status are lower in times of high unemployment and if many asylum seekers from the same country have already applied. This suggests that social factors are influencing individual asylum decisions in a way that must surely be inappropriate.

In the USA, there has been a very large-scale audit of the decision-making process and in the subsequent report (appropriately entitled “Refugee Roulette”), the authors publish some of their findings (Ramji-Nogales, Schoenholtz, & Schrag, 2007).

The authors were provided with data from all eight regional offices covering 928 US asylum officers (first-line decision-makers) over 6 years. Across all eight regions, the variation in mean grant rates in Chinese cases (to take one example) ranged from under 20% in one region to over 70% in another. However, averages like these hide the extent of the variation. In one of these regions, grant rates by individual officers varied between 0 and 68%; one officer had considered 273 Chinese cases and had not granted a single asylum claim. There was even greater variation in the decisions made by judges. For example, in Los Angeles, 22 experienced judges assessing similar Chinese cases made remarkably different decisions. One judge accepted 9% (of 117 cases) and another 81% (of 118 cases). The authors found that quality of legal representation had a profound effect on outcome. Whereas those asylum applicants who were unrepresented had a 16.3% chance of success, those routinely represented had a 45.6% chance of success and those represented by a specialist clinic had an 89% chance of success. Asylum applicants represented pro bono by large law firms had a success rate of about 96%.

Curiously the judge's gender also had a bearing on outcome; female judges granting 53.8% and male judges 37.3% of cases. An asylum applicant assigned by chance to a female judge therefore had a 44% better chance of success. Previous experience of the judges—government service, military, non-profit organisations, academia, and private practice—all had a substantial effect on case outcomes. In brief, factors that should have had no impact on outcome of individual applications were surprisingly important.

No similar large-scale audit of UK practice has been undertaken. However, some data are available. In the year to end March 2015, there were 25,020 asylum applications in the UK. Initial decisions appropriately varied by country of origin; 85% of initial decisions for nationals of Eritrea and Syria were grants compared with 22% for Pakistani nationals. Overall, 40% (10,346) of decisions were grants either of asylum or an alternative form of protection. There were 11,067 appeals received in the year to March 2015. Of cases heard, 28% of appeals were allowed, once again suggesting that these are not easy or reliable decisions.

In a recent publication (Herlihy & Turner, 2015), some of the psychological evidence has been reviewed (also see Herlihy, Jobson, & Turner, 2012 and Herlihy & Turner, 2013). There is now an increasing body of scientific evidence relevant to this issue, including work looking at the assumptions recorded by Judges in their asylum decisions (Herlihy, Gleeson, & Turner, 2010) and work looking directly at the psychological processes at play in those seeking asylum. This literature suggests not only that there may be marked uncertainty in how to reach the correct determination but also that there is often the potential for bias *against* the person genuinely fleeing from trauma and persecution. Bögner, Herlihy, and Brewin (2007) investigated the impact of psychological state on Home Office interviews. Those with a history of having been exposed to sexual violence reported more difficulties in disclosing personal information during Home Office interviews, were more likely to dissociate in the interview, and scored significantly higher on measures of PTSD and shame, suggesting the potential for a positive bias in this procedure against survivors of sexual violence. There is now evidence indicating that it is incorrect for judges to assume that discrepancies in narratives given at different times necessarily indicate fabrication (a relatively common assumption made in decisions). The data indicate that discrepancies are more common in those with higher scores on a measure of PTSD symptomatology, especially when there are long delays between interviews, thus potentially disadvantaging the traumatised asylum seeker (Herlihy, Scragg, & Turner, 2002). Moreover, there is work looking at overgeneral memory retrieval (Graham, Herlihy, & Brewin, 2014) showing that in a sample of asylum seekers, those with PTSD and depression recalled fewer specific memories, making it harder to present their claim. It is not an unreasonable expectation that those who have been through torture or similar extreme violence will have both higher trauma symptoms and also secrets (perhaps betrayals or aspects of the experience) which they will try to hide; in an analogue study, Rogers, Fox, and Herlihy (2014) report on how behavioural cues are interpreted in assessing credibility, finding, for example, that where an actor presented with mixed trauma and deception behaviours, they were rated less credible than a comparison condition in which an actor presented pure deception behaviour.

It is now essential for there to be collection and dissemination of scientific evidence concerning asylum seekers and their testimonies. It is no longer acceptable to rely on common sense, when there is better evidence available. Although experienced advocates do have more knowledge of the emotional reactions experienced by refugees, this is still limited and not a substitute for a full psychological assessment in appropriate cases (Wilson-Shaw, Pistrang, & Herlihy, 2012).

Finally, no paper on this topic would be complete without some reference to treatment facilities. In contrast to many other subgroups with PTSD, this has often been seen as too complex for scientific scrutiny or even unethical to research. Droždek (2015) makes a good case for multimodal approaches in treatment and certainly cultural sensitivity has to be an important element in any successful programme. Standard trauma-focused treatments probably have their role (e.g., Acarturk et al., 2015), although we lack the range of scientific data on treatment efficacy available for other victim groups. Narrative Exposure Therapy (Schauer, Neuner, & Elbert, 2011) perhaps brings together some of these treatment approaches. The main point for the purpose of the present article is that refugees should be able to avail themselves of treatment and rehabilitation services just as anyone else, and that it should be just as important to build up the evidence base.

## Conclusions

Relatively few asylum seekers find their way to Western Europe. UNHCR estimates that in 2014, Turkey, Pakistan, Lebanon, and Iran hosted more than 5.2 million refugees between them. If these countries are expected to respond to need on such a scale, it sets in context the numbers coming to Europe. Policies designed to deter entry serve to prevent many who would otherwise be accepted as refugees from travelling to Europe, and for those who do travel, they probably help to support the economic basis for people smuggling and stand to increase dramatically the risks involved for those in transit. Finally, for those who do arrive, the decision-making process itself is probably flawed, with very wide discrepancies between decision-makers (“refugee roulette”) and potential biases against those with a history of traumatisation.

This paper does not set out to provide simple answers to what is self-evidently a complex problem. It does argue for a detailed systemic data-driven analysis and not a flight into naive political rhetoric. The evidence presented here probably favours various approaches.

First, we need to recognise the large growth of migration in the world as a whole and set out to understand the reasons for this. International aid (as a broad category, including financial aid, trade, and development) can and should be used to support economic growth in poorer parts of the world. Policies can also support nations who are starting to make their own shift away from autocracy; implementing human rights policies, supported by an international legal framework, is likely to reduce the pressure to leave countries. Western military action, for example, in the Middle East, has often had its own adverse effects and the costs of these should be recognised from the outset, with appropriate steps built in to foster successful nation-building. Most refugees find themselves in camps in neighbouring countries and financial support for this local refugee response should be supported as a (likely) cost-effective way of making life more bearable there (a humanitarian impulse) and thereby also reducing the drive to move on to the West (a partialist political impulse).

Second, we need to recognise the weaknesses in the policies and systems we have set up in the West. This will be tough because public opinion currently is not always supportive. Nonetheless, we do need to start moving the argument away from “bogus asylum seekers” or “swarms of migrants” and recognise that many of those we prevent from arriving here, from countries like Syria and Eritrea, would receive humanitarian protection had they been able to travel. Current policy is often designed simply to stop migrants accessing our decision-making processes. This applies at the EU's external borders (e.g., with the new fence on the Hungarian border and with operation Triton in the Mediterranean) as well as at the EU's internal borders (e.g., at Calais and even occasionally within the Schengen area). As with other policies of prohibition, this approach stands to favour the commercial market in people smuggling. Perhaps there is no political will in European countries to take more refugees, but there is absolutely no reason why the debate should not be open and honest. Current evidence suggests that there need to be further reforms to the Dublin regulation and more coordination of refugee reception and other policies across the European Union.

Finally, we must recognise the weaknesses of the decision-making process itself. There is a pressing need for more research and for the implementation of that body of psychological knowledge that exists concerning testimony evidence and trauma. Our courts should set out to be examples of best practice in a way that they have probably not yet achieved. If we compromise on that principle with regard to asylum seekers, we stand to compromise on fundamental principles of fairness and justice for us all.
